# Editorial Notes, News and Miscellany

**Published:** 1913-09

**Authors:** 


### Editorial Notes, News and Miscellany.

DR. L. B. TENNEY has removed from Elysian Fields, Texas, to Troupe, Texas.

THE PICTURE OF DR. WYETH in this issue was intended for the August issue, to accompany a personal sketch, and a write up of the great Polyclinic; but by some unaccountable and inexcusable negligence in the bindery, it was left out.

---

Dr. A. C. Oliver of Cass county who had just been elected to the Senate to fill a vacancy, has been appointed Superintendent of the Confederate Soldiers' Home, vice Lyles resigned. I "wish him joy;" I would not have it for a million a minute."

---

Dr. Augustus Maverick a popular and prosperous young physician of San Antonio, was shot and killed in his home in that city one night recently, by a negro who had entered the house for felonious purposes. The negro will be hanged on the 26th of this month.

---

Biological Products and How to use Them, is the title of a neat booklet just issued by the Abbott Alkaloidal Co., Chicago. They will be pleased to mail free of cost a copy to any inquirer who mentions this notice. Also a copy of their new Therapeutic Price List.

---

Congressman Herman A. Metz, of the 10th New York District, president of the Farbwerke-Hoechst Company, importers of Salvarsan, Neosalvarsan, Novocain, etc., has been designated by the Demcoratic party for controller of the City of New York. He served in this position with the greatest credit to himself from 1906 to 1910, and during that period he placed the finances of the city upon a firm foundation.

---

Personal.—Dr. Isaac E. Clark, of Schulenburg, now Senator Clark, distinguished himself in the recent fight in the Senate over the confirmation of the Governor's reappointment of the three Penitentiary Commissioners. A special committee with the Lieutenant Governor, as chairman, had investigated the management of penitentiary affairs under these commissioners and had reported a deficit or shortage of over a million dollars which the Legislature had to make good and the taxpayers pay, "due to negligence, incompetence and gross mismanagement," and yet the Governor had sent in their names for reappointment! If it had not been for Clark, it is highly probable they would have been reappointed, "for political reasons." A large minority of the Senators would condone it, as no stealing was actually shown. Clark will not stand for any such, and he bucked against the leaders and really prevented the outrage. The *Tribune* says of it:

"The fight on the board had been vigorously made by Senator Clark of Schulenburg, who is one of those old-fashioned, sturdy, upright, vigorous characters who made America what it is. While an anti on liquor legislation, he is above all of sterling integrity, with no patience for inefficiency and a hatred of wrong-doing. Perhaps Texas is more indebted to his uncompromising demand for a new deal than to any other member of the senate."

All honors to Dr. Clark.

